# In silico exploration of anti-prostate cancer compounds from differential expressed genes

**DOI:** 10.1186/s12894-024-01521-9

**Published:** 2024-07-03

**Authors:** Basiru Olaitan Ajiboye, Toluwase Hezekiah Fatoki, Olamilekan Ganiu Akinola, Kazeem Olasunkanmi Ajeigbe, Abraham Fisayo Bamisaye, Eva-María Domínguez-Martín, Patricia Rijo, Babatunji Emmanuel Oyinloye

**Affiliations:** 1 Phytomedicine and Molecular Toxicology Research Laboratory, Department of Biochemistry, Federal University Oye-Ekiti, Oye-Ekiti, Ekiti State Nigeria; 2 Applied Bioinformatics Research Laboratory, Department of Biochemistry, Federal University Oye-Ekiti, Oye-Ekiti, Ekiti State Nigeria; 3 Department of Physiology, Faculty of Basic Medical Sciences, Federal University Oye-Ekiti, Oye-Ekiti, Ekiti State Nigeria; 4 Department of Biochemistry, Federal University Oye-Ekiti, Oye-Ekiti, Ekiti State Nigeria; 5 CBIOS—Universidade Lusófona’s Research Center for Biosciences & Health Technologies, Lusófona University, Campo Grande 376, Lisbon, 1749-024 Portugal; 6 Facultad de Farmacia, Departamento de Ciencias Biomédicas (Área de Farmacología), Universidad de Alcalá de Henares, Nuevos Agentes Antitumorales, Acción Tóxica Sobre Células Leucémicas, Ctra. Madrid-Barcelona km. 33,600, Alcalá de Henares, Madrid, 28805 España; 7 Phytomedicine, Biochemical Toxicology and Biotechnology Research Laboratories, Department of Biochemistry, College of Sciences, Afe Babalola University, Ado-Ekiti, Nigeria; 8 Biotechnology and Structural Biology (BSB) Group, Department of Biochemistry and Microbiology, University of Zululand, KwaDlangezwa, 3886 South Africa

**Keywords:** Prostate cancer, DEGs, ADMET, Molecular targets, Gene network, Molecular docking, Molecular dynamic simulation

## Abstract

Prostate cancer (PCa) is a complex and biologically diverse disease with no curative treatment options at present. This study aims to utilize computational methods to explore potential anti-PCa compounds based on differentially expressed genes (DEGs), with the goal of identifying novel therapeutic indications or repurposing existing drugs. The methods employed in this study include DEGs-to-drug prediction, pharmacokinetics prediction, target prediction, network analysis, and molecular docking. The findings revealed a total of 79 upregulated DEGs and 110 downregulated DEGs in PCa, which were used to identify drug compounds capable of reversing the dysregulated conditions (dexverapamil, emetine, parthenolide, dobutamine, terfenadine, pimozide, mefloquine, ellipticine, and trifluoperazine) at a threshold probability of 20% on several molecular targets, such as serotonin receptors 2a/2b/2c, HERG protein, adrenergic receptors alpha-1a/2a, dopamine D3 receptor, inducible nitric oxide synthase (iNOS), epidermal growth factor receptor erbB1 (EGFR), tyrosine-protein kinases, and C-C chemokine receptor type 5 (CCR5). Molecular docking analysis revealed that terfenadine binding to inducible nitric oxide synthase (-7.833 kcal.mol^−1^) and pimozide binding to HERG (-7.636 kcal.mol^−1^). Overall, binding energy ΔG^bind^ (Total) at 0 ns was lower than that of 100 ns for both the Terfenadine-iNOS complex (-101.707 to -103.302 kcal.mol^−1^) and Ellipticine-TOPIIα complex (-42.229 to -58.780 kcal.mol^−1^). In conclusion, this study provides insight on molecular targets that could possibly contribute to the molecular mechanisms underlying PCa. Further preclinical and clinical studies are required to validate the therapeutic effectiveness of these identified drugs in PCa disease.

## Introduction

Cancer is a condition characterized by genetic or epigenetic changes in somatic cells, leading to abnormal cell growth that can potentially spread to other parts of the body. These abnormal growths, known as neoplasms or tumors, can manifest as localized masses or diffuse distributions [1, 2]. Globally, cancer is a major cause of mortality, accounting for approximately 9.6 million deaths, and it is projected that around 15 million new cases will be diagnosed as the world population reaches 7.5 billion by 2020 [3]. Furthermore, there is an anticipated annual increase of approximately 420 million new cancer cases by 2025, indicating a rising incidence of cancer over the coming years [2].

The causes of cancer are attributed to both internal factors (such as inherited mutations, hormonal imbalances, and immune conditions) and external or environment factors (such as tobacco use, diet, exposure to radiation, and infectious agents). Several modifiable risk factors contribute significantly to the development of cancer, including tobacco use, being overweight or obese, leading a sedentary lifestyle, excessive alcohol consumption, exposure to certain infections, outdoor and indoor air pollution, and exposure to occupational carcinogens [4].

Prostate cancer (PCa) is a complex and diverse disease with multiple biological characteristics [5]. PCa is the second most commonly diagnosed cancer and the fifth leading cause of cancer-related deaths among men worldwide [5, 6]. In 2020, 1,414,000 new cases of PCa were estimated with 375,304 deaths attributed to the disease [6]. Moreover, it has been forecasted that by 2040, 2.43 million new cases of PCa with 740,000 deaths worldwide will be recorded [7, 8]. PCa ranks as the most frequently diagnosed cancer in 112 countries and is the leading cause of cancer mortality in 48 countries [9]. While data on PCa incidence and mortality in Africa is limited, with specific information available for countries such as Mauritius, Zimbabwe, and South Africa, the incidence of PCa in Africa and Asia tends to be lower compared to other regions [6].

PCa predominantly affects middle-aged men, typically between the ages of 45 and 60, and it is a leading cause of cancer-related deaths in Western countries [10]. Diagnosis of PCa commonly involves techniques such as prostate biopsy, prostate-specific antigen (PSA) testing, digital rectal examination, magnetic resonance imaging (MRI), and health screenings [5]. Risk factors associated with PCa include family history, ethnicity, age, obesity, and environmental factors. PCa exhibits heterogeneity both in terms of epidemiology and genetics [5]. The interplay between genetics, environmental influences, and social factors contributes to race-specific variations in PCa survival rates, leading to observed differences in the epidemiology of the disease across different countries [11]. It has been noted that almost all PCa often acquire resistance to become castration-resistant prostate cancer (CRPC) based on dysfunctional androgen receptor activities due to mutations, loss of expression or other hormonal receptors [12]. Effective treatment of CRPC is still challenging and ongoing research efforts have provided drugs that only prolong overall survival of CRPC patients by few months [12].

PCa treatment has seen significant advancements in recent years, with ongoing research aimed at improving patient outcomes and quality of life. According to Chen and Zhao [13], and Varaprasad et al. [8], the current state of PCa treatment includes (A) Standard treatments such as (i) Surgery (Radical prostatectomy) which involves the surgical removal of the prostate gland and surrounding tissue. (ii) Radiation therapy (External beam radiation therapy (EBRT) and brachytherapy (internal radiation)) which are used to target and kill cancer cells in the prostate. (iii) Hormone therapy which also known as androgen deprivation therapy (ADT), reduces levels of male hormones that can stimulate cancer growth. (iv) Chemotherapy (such as docetaxel and cabazitaxel drug) is used primarily for advanced PCa that is resistant to hormone therapy. (v) Targeted therapy (such as abiraterone and enzalutamide drug) target specific pathways involved in PCa growth. (vi) Immunotherapy (such as Sipuleucel-T) is designed to stimulate the body’s immune system to attack cancer cells. (B) Emerging treatments include (i) PARP Inhibitors (such as Olaparib drug) are used for patients with specific genetic mutations. (ii) Radiopharmaceuticals (such as radium-223 dichloride) target cancer cells with radiation while minimizing damage to surrounding tissues. (iii) Advanced immunotherapies (such as checkpoint inhibitors and personalized cancer vaccines) and nanotherapies.

The existing treatment options for PCa are not curative, and it has been recognized that a single targeted therapy is insufficient to significantly impact the progression of PCa [14]. As a result, the exploration of medicinal plants as alternative source for PCa treatment is being investigated due to their poly-pharmacological effects. Combined therapies involving current treatment options for PCa have shown promise in extending patients’ lifespans and suppressing tumor growth. Additionally, the repurposing of existing drugs such as metformin, naftopidil, triclosan, niclosamide, and glipizide for the treatment of PCa has been proposed [15, 16].

Understanding the molecular events involved in the development of metastatic PCa has the potential to identify biological determinants that can aid in prognosis and development of more effective therapies [17]. Differentially expressed genes (DEGs) analysis in PCa offers valuable insights by identifying genes with altered expression levels, highlighting potential key players in the disease, though with some inherent limitations. The rationale of this present work was based on the fact that computational analyses of DEGs in metastatic PCa allows comprehensive understanding of molecular changes, and that identification of drugs that modulate these genes toward normal expression levels could pave the way for targeted therapies. Computational techniques have been instrumental in drug repurposing, where existing drugs are tested for new therapeutic uses. By analyzing DEGs, it has been possible to predict how well-known drugs might affect new targets, and this approach has led to the selection of several compounds as promising candidates for treating diseases such as COVID-19 and cancer [15, 16, 18].

Computational methods have revolutionized the field of drug discovery and repurposing, particularly for complex diseases like PCa. These methods leverage advanced algorithms, machine learning, and big data analytics to accelerate and refine the drug development process. Key computational approaches include virtual screening and molecular docking, pharmacophore modeling, quantitative structure-activity relationship (QSAR) models, genomic and proteomic data integration, artificial intelligence, deep learning and machine learning in clinical trials, and drug repurposing [19, 20]. The integration of advanced computational methods into PCa research is accelerating the discovery of new treatments and the repurposing of existing drugs. These technologies offer promising avenues for improving patient outcomes by enabling more precise and personalized approaches to therapy. As computational power and algorithm sophistication continue to advance, the potential for breakthroughs in PCa treatment grows, offering hope for more effective and targeted interventions in the future. Overall, the use of computational techniques with DEGs has revolutionized drug discovery by making it faster, more cost-effective, and more precise, thereby improving the development of targeted therapies and personalized medicine. Therefore, this study aims to computationally identify compounds that could be used as novel agents or repurposed for the treatment of PCa by exploring differentially expressed genes (DEGs).

## Materials and methods

### Gene expression dataset

The gene expression dataset of PCa generated and published by Chandran et al. [17] were used for this study. The dataset served as the basis for the analysis and exploration conducted in this study.

### DEGs network analysis

The DEGs network analyses, consisting of transcription factor, protein-protein interaction and kinase enrichments, were conducted using the eXpression2Kinases (X2K) Web server at https://maayanlab.cloud/X2K/ [21]. The X2K Web server provided a platform to explore and interpret the gene expression data in the context of transcriptional regulation, protein interactions, and kinase signalling, thereby offering valuable insights into the molecular mechanisms underlying the observed differentially expressed genes in the context of PCa.

### Ligand discovery analysis

In the ligand discovery analysis, Expression2Kinases (X2K) software [22] was used to determine the top 10 drugs capable of reversing the expression of both upregulated and downregulated differentially expressed genes [18]. The software was configured with default settings and human was selected as the organism of interest. The drug prediction module of X2K utilizes the Connectivity Map database to rank drugs based on their potential to induce or reverse the expression of DEGs [22]. This feature is particularly useful for discovering drugs that might modulate specific signaling pathways identified in the gene expression analysis.

### In silico ADME/T prediction

The ligands identified in the previous steps were searched in the PubChem database (PubChem CIDs: 65,808; 10,219; 7,251,185; 36,811; 5405; 16,362; 4046; 3213; and 5566) and their SMILES representations were obtained. The SMILES were used for in silico ADME/T (Absorption, Distribution, Metabolism, Excretion, and Toxicity) prediction. Firstly, SwissADME webserver, accessible at www.swissadme.ch [23] was used to predict the ADME. Furthermore, pkCSM webserver, accessible at http://biosig.unimelb.edu.au/pkcsm/ [24], was employed for ADMET analysis. SwissADME is a free web tool designed to evaluate the pharmacokinetics, drug-likeness, and medicinal chemistry friendliness of small molecules. pkCSM is a computational tool used to predict pharmacokinetic properties and toxicity of small molecules in drug discovery. It employs graph-based signatures to model the relationships between molecular structures and their biological effects.

### In silico target prediction

The SMILES were used for in silico target prediction on SwissTargetPrediction webserver (http://www.swisstargetprediction.ch/). In the analysis, *Homo sapiens* was selected as the target organism [25]. SwissTargetPrediction is an online tool designed to predict the biological targets of small molecules. It utilizes a combination of 2D and 3D similarity measures to compare a query molecule against a database of known ligands and their targets. This approach helps identify potential protein targets for drug discovery and development, aiding researchers in understanding the mechanisms of action of compounds and in repurposing existing drugs​.

### Molecular docking studies

The molecular docking studies were conducted following the methodology described by Fatoki et al. [26]. Initially, the three-dimensional structures of the most probable proteins were obtained as AlphaFold pdb format through the UniProt database (UniProt IDs: Q12809; P35462; P35228; P00533, P06241; P23415; and P11388). The structure ligands in SMILES were converted to mol format using ACDLab/Chemsketch software. Subsequently, PyMol software was utilized for the conversion of ligand files from .mol to .pdb format. Both the ligands and the protein were prepared for docking using AutoDock Tools (ADT) v1.5.6 [27] with default settings, and the output file was saved in pdbqt format. The molecular docking experiments were performed using the AutoDock Vina v1.2.3 [28, 29]. Following the docking process, the interactions involved in the binding of the ligands to the target protein were analyzed and visualized using ezLigPlot webserver [30]. AutoDock Vina is an open-source molecular docking software with improves accuracy and speed of docking simulations through an efficient optimization algorithm and a scoring function that estimates the binding affinity of ligands to their targets. This tool is widely utilized in computational drug discovery and structural biology to aid in the identification and optimization of potential drug candidates​.

### Molecular dynamics simulation

MD simulations were conducted using Desmond v3.6, in a Schrödinger LLC software v2021-1 [26, 31, 32]. Desmond is a high-performance molecular dynamics (MD) simulation software developed by D. E. Shaw Research. It is widely used in computational chemistry and drug discovery due to its speed and accuracy. Briefly, the initial stage of protein and ligand complexes for molecular dynamics simulation were obtained from docking studies. The protein–ligand complexes were preprocessed using maestro’s protein preparation wizard, which also included optimization and minimization of complexes. All systems were prepared by the System Builder tool. Solvent Model with an orthorhombic box was selected as TIP3P (Transferable Intermolecular Interaction Potential 3 Points). The Optimized Potential for Liquid Simulations (OPLS)-2005 force field was used in the simulation [33]. The models were made neutral by adding counter ions 0.15 M NaCl to mimic the physiological conditions [34]. The NPT ensemble (Isothermal-Isobaric: moles (N), pressure (P), and temperature (T) are conserved) with 300 K temperature and 1 atm pressure) was select for complete simulation. The models were relaxed before the simulation, and full system simulation was performed for 100 ns with trajectories saved every 100 ps. The post-simulation analyses of the trajectories were done to determine the root-mean-square deviation (RMSD), root-mean-square fluctuation (RMSF), and protein-ligand interaction profile. Also, prime molecular mechanics/generalized Born surface area (MMGBSA) was used to evaluate the binding free energy [26, 35, 36], as follows:

- MMGBSA ΔG^bind^ = ΔG^complex^ - ΔG^protein^ - ΔG^ligand^.
- MMGBSA ΔG^bind^ = ΔG^Coulomb^ + ΔG^Covalent^ + ΔG^Hbond^ + ΔG^Lipo^ + ΔG^Packing^ + ΔG^SolvGB^ + ΔG^vdW^.

where ΔG^bind^ is the total Prime energy, Hbond denote hydrogen bonding energy, Lipo is lipophilic energy, Packing represents pi-pi packing correction. SolvGB is generalized Born electrostatic solvation energy, and vdW is Van der Waals energy.

## Results

PCa is a leading cause of cancer-related mortality among men worldwide, presenting a significant public health challenge. In this study, DEG data was integrated with in silico techniques to offer a promising avenue for discovering new anti-PCa compounds. By focusing on genes that are specifically altered in PCa, we identify compounds that selectively target these pathways.

**Fig. 1:**
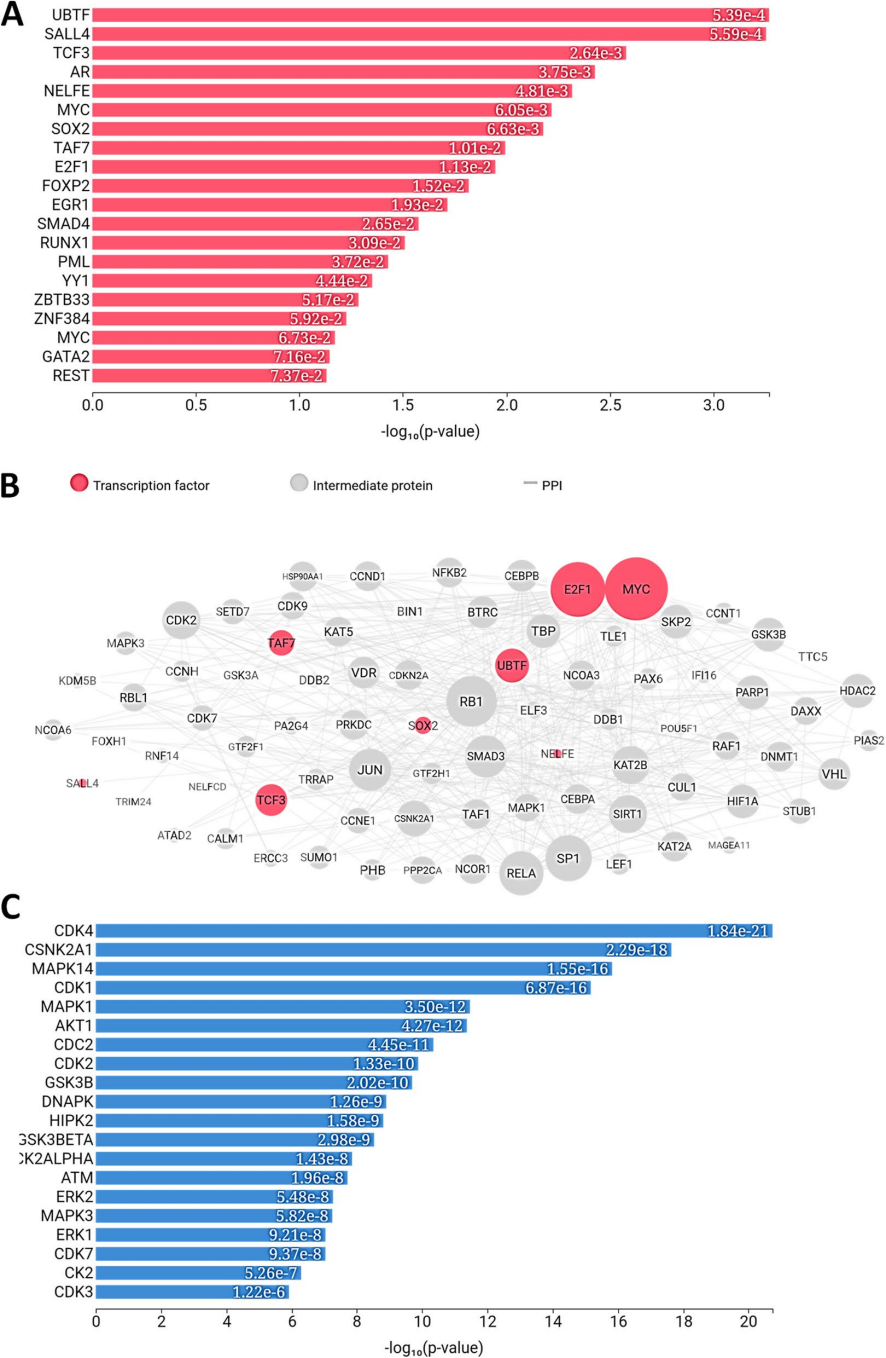
Enrichment analysis of upregulated DEGs showing (**A**) Transcription factor (**B**) protein-protein interaction and (**C**) kinases

**Fig. 2:**
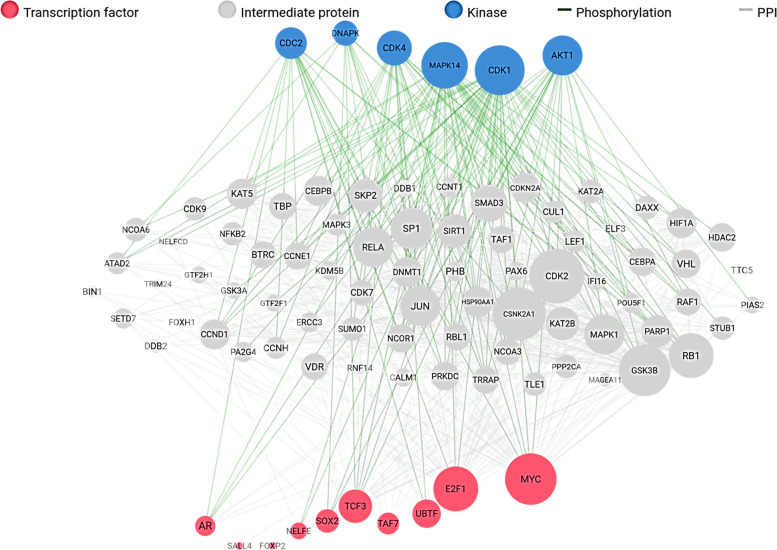
Overall enrichment analysis network of up-regulated DEGs

**Fig. 3:**
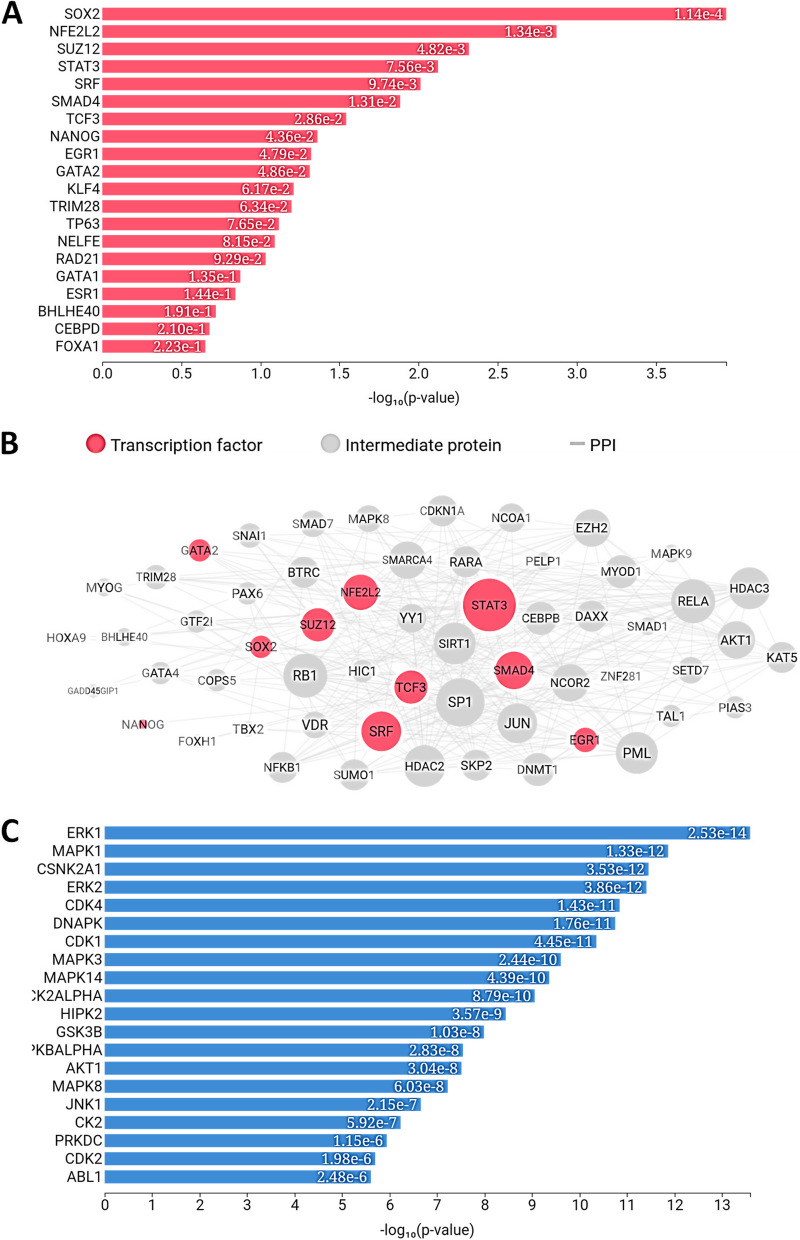
Enrichment analysis of down-regulated DEGs enrichment showing (**A**) Transcription factor (**B**) protein-protein interaction and (**C**) kinases

**Fig. 4:**
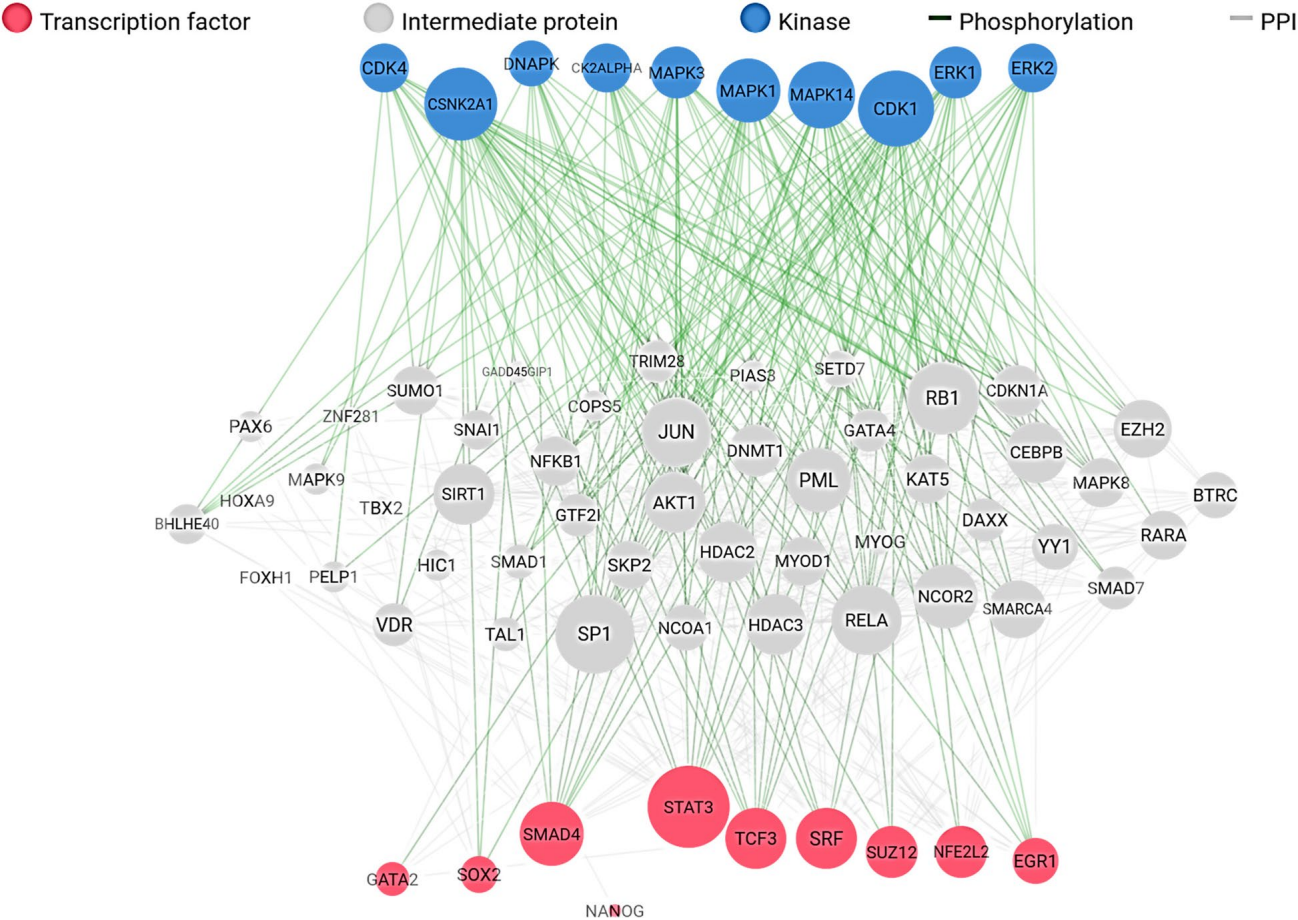
Overall enrichment analysis network of down-regulated DEGs

A total of 79 upregulated DEGs and 110 downregulated DEGs in PCa (PCa) were analysed. The results of gene network analyses for the upregulated genes yielded enriched transcription factors (POU3F2, CLOCK, CTNNB1, HNF4A, E2F1, CREB1, NANOG, SOX2, MYC and WT1), and enriched kinases (PRKG1, ERBB4, RNASEL, DAPK2, DDR1, NTRK2, AXL, PKN2, CDC2 and SRPK1) as shown in Fig. 1. The results of overall expression network for the upregulated DEGs showed the enriched kinases (MAPK14, CDK1, AKT1, CDK4, CDC2, and DNAPK), transcription factor (E2F1, MYC, UBTF, TAF7, TCF3, SOX2, FOXP2, SALL4, and AR) while the enriched intermediate proteins include GSK3B, RELA, CDK2, MAPK1, PARP1, JUN, SIRT1, and RB1, as shown in Fig. 2. The results of gene network analyses for the downregulated genes, enriched transcription factors are CUX1, SOX2, POU5F1, AR, ESR2, ESR1, and PAX3-FKHR, and enriched kinases are PRKG2, RPS6KA6, PKN1, ACVR2A, PRKG1, FRK, EPHB1, ROCK2, EPHB6, and ACVR2B as shown in Fig. 3 The results of overall expression network for the downregulated DEGs showed the enriched kinases (MAPK14, MAPK1, MAPK13, CSNK2A1, CDK1, CDK4, ERK1, ERK2, and DNAPK), transcription factor (STAT3, TCF3, SRF, SUZ12, NFE2L2, SMAD4, SOX2, GATA2 and EGR1) while the enriched intermediate proteins include SP1, GSK3B, RELA, JUN, RB1, HDAC3, SIRT1, and NCOR2 as shown in Fig. 4.

**Fig. 5:**
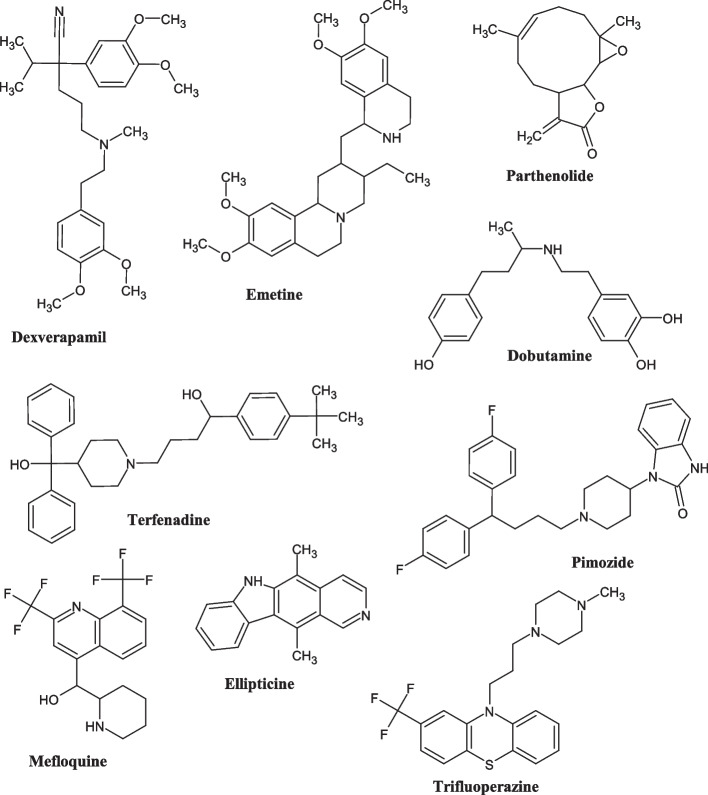
Chemical structures of the investigated compounds

The results of ligand discovery showed the top drugs that could reverse upregulated genes in PCa, which are talampicillin, dexverapamil, homosalate, emetine, gemfibrozil, parthenolide, cephaeline, hesperidin, cycloheximide, dobutamine, ginkgolide A, kanamycin, and diclofenamide; while the drugs that could reverse downregulated genes in PCa are: terfenadine, camptothecin, menadione, pimozide, mefloquine, digoxigenin, strophanthidin, nitrofurantoin, felodipine, anisomycin, ellipticine, trichostatin A, propofol, and trifluoperazine. The chemical structures of nine drug compounds selected for further investigation in this study are shown in Fig. 5.

The predicted absorption, distribution, metabolism and excretion (ADME) or pharmacokinetics of the nine selected drugs indicate that all the selected nine have low GIA, only Pimozide and Mefloquine are not BBB permeants, only Emetine and Parthenolide are not inhibitors of cytochrome P450 (CYPs) type CYP1A2, CYP2C19, CYP2C9, CYP2D6, and CYP3A4. Also, only Parthenolide is not a substrate of p-glycoprotein, and only Terfenadine and Pimozide are poorly soluble, as shown in Table 1. Furthermore, ADMET results in Table 2 indicate that Parthenolide, has the highest intestinal absorption followed by Ellipticine and Dexverapamil. Also, Parthenolide, Dobutamine and Ellipticine are not inhibitors of p-glycoprotein I and II. The toxicity results showed that only Parthenolide, Pimozide and Ellipticine had AMES toxicity potential, only Terfenadine and Pimozide are not a potential inhibitor of hERG I and II, while Dexverapamil, Emetine, Parthenolide, Terfenadine, and Ellipticine have no potential hepatotoxicity. In the subsequent analyses, gemfibrozil and camptothecin were ignored.

**Table 1:** Ligands ADME properties using SWISSADME webserver

SN	Ligands	PubChem CID	Predicted ADME properties												
			MW	MR	TPSA (Å^2^)	Log *P*	ESOL Log S	ESOL Class	GIA	BBB permeant	*P*-gp	CYPs Inhibitor	Log Kp (cm/s)	BS	SA
1	Dexverapamil	65,808	454.6	132.46	63.95	4.45	-4.46	Moderately soluble	High	Yes	Yes	CYP2D6, CYP3A4	-6.38	0.55	3.75
2	Emetine	10,219	480.64	147.05	52.19	4.19	-5.6	Moderately soluble	High	Yes	Yes	-	-5.87	0.55	4.87
3	Parthenolide	7,251,185	248.32	69.34	38.83	2.64	-2.85	Soluble	High	Yes	No	-	-6.15	0.55	4.64
4	Dobutamine	36,811	301.38	88.8	72.72	2.91	-3.81	Soluble	High	Yes	Yes	CYP2D6, CYP3A4	-5.7	0.55	2.43
5	Terfenadine	5405	471.67	149.82	43.7	5.73	-6.69	Poorly soluble	High	Yes	Yes	CYP2D6	-4.51	0.55	3.96
6	Pimozide	16,362	461.55	135.86	41.03	5.72	-6.67	Poorly soluble	High	No	Yes	CYP2C19, CYP2D6	-4.64	0.55	3.2
7	Mefloquine	4046	378.31	86.51	45.15	4.13	-4.49	Moderately soluble	High	No	Yes	CYP2D6, CYP3A4	-6.04	0.55	3.25
8	Ellipticine	3213	246.31	81.04	28.68	3.92	-5.05	Moderately soluble	High	Yes	Yes	CYP1A2, CYP2C19, CYP2D6, CYP3A4	-4.39	0.55	1.6
9	Trifluoperazine	5566	407.5	118.1	35.02	4.47	-5.52	Moderately soluble	High	Yes	Yes	CYP1A2, CYP2D6	-5.21	0.55	3.47

*Physicochemical properties*: *MW* Molecular weight, *MR* Molar Refractivity, *TPSA *Total polar surface area,  *Lipophilicity*: Consensus Log P. *Water Solubility*: ESOL Log S, ESOL Class. *Pharmacokinetics*: *GIA *Gastrointestinal absorption, *BBB* Blood-brain barrier, P-glycoprotein (P-gp) substrate, Inhibition of Cytochrome P450 (CYPs) type CYP1A2, CYP2C19, CYP2C9, CYP2D6, and CYP3A4, Skin permeation (Log Kp). *Druglikeness*: *BS *Bioavailability Score, *Medicinal Chemistry*: *SA *Synthetic accessibility

**Table 2:** Ligand ADMET properties using pkCSM webserver

ADMET		LIGANDS								
Type	Properties	A	B	C	D	E	F	G	H	I
Absorption	Water solubility (log mol/L)	-5.421	-3.666	-3.161	-3.169	-4.286	-2.899	-4.874	-5.049	-4.837
	Caco-2 permeability (log Papp in 10 cm/s)	0.547	0.751	1.71	0.883	1.014	0.121	1.446	1.407	1.009
	Intestinal absorption (human) (% Absorbed)	92.836	91.032	97.599	86.587	89.765	84.897	85.961	95.756	90.906
	Skin Permeability (log Kp)	-2.763	-2.798	-3.278	-2.735	-2.735	-2.735	-2.96	-2.737	-2.73
	P-glycoprotein substrate	Yes	Yes	No	Yes	Yes	Yes	Yes	Yes	Yes
	P-glycoprotein I inhibitor	Yes	Yes	No	No	Yes	Yes	Yes	No	Yes
	P-glycoprotein II inhibitor	Yes	Yes	No	No	Yes	Yes	Yes	No	Yes
Distribution	VDss (human) (log L/kg)	0.931	1.596	0.291	1.738	0.529	0.616	0.83	0.072	2.223
	Fraction unbound (human)	0.025	0.204	0.45	0.423	0	0.185	0.193	0.136	0.041
	BBB permeability (log BB)	-0.647	-0.394	0.444	-0.738	0.222	0.004	0.488	0.414	0.847
	CNS permeability (log PS)	-2.484	-2.067	-3.007	-2.519	-1.342	0.487	-2.675	-1.209	-1.541
Metabolism	CYP2D6 substrate	No	Yes	No	No	Yes	No	No	No	Yes
	CYP3A4 substrate	Yes	Yes	No	Yes	Yes	Yes	Yes	Yes	Yes
	CYP1A2 inhibitor	No	No	No	No	Yes	Yes	Yes	Yes	Yes
	CYP2C19 inhibitor	No	Yes	No	No	Yes	No	No	Yes	Yes
	CYP2C9 inhibitor	No	No	No	No	No	Yes	No	No	No
	CYP2D6 inhibitor	Yes	Yes	No	Yes	Yes	Yes	No	No	Yes
	CYP3A4 inhibitor	Yes	No	No	No	No	Yes	No	Yes	Yes
Excretion	Total Clearance (log ml/min/kg)	1.072	0.993	1.162	1.132	0.718	0.631	0.43	0.535	0.385
	Renal OCT2 substrate	Yes	No	Yes	No	No	Yes	Yes	No	No
Toxicity	AMES toxicity	No	No	Yes	No	No	Yes	No	Yes	No
	Max. tolerated dose (human) (log mg/kg/day)	-0.181	-0.019	0.306	0.105	0.41	0.107	-0.283	0.288	0.104
	hERG I inhibitor	No	No	No	No	Yes	Yes	No	No	No
	hERG II inhibitor	Yes	Yes	No	Yes	Yes	Yes	Yes	No	Yes
	Oral Rat Acute Toxicity (LD50) (mol/kg)	2.973	2.793	2.096	2.951	2.252	2.442	2.926	2.236	2.751
	Oral Rat Chronic Toxicity (LOAEL) (log mg/kg_bw/day)	1.309	0.674	1.592	1.101	1.063	1.088	0.473	1.506	0.851
	Hepatotoxicity	No	No	No	Yes	No	Yes	Yes	No	Yes
	Skin Sensitisation	No	No	Yes	No	No	No	No	No	No
	*T. Pyriformis* toxicity (log ug/L)	0.667	0.327	0.46	0.305	0.287	0.285	1.186	0.489	1.057
	Minnow toxicity (log mM)	-1.947	-0.825	1.582	2.009	0.379	2.339	0.913	-1.289	4.016

*Legend:* A: Dexverapamil. B: Emetine. C: Parthenolide. D: Dobutamine. E: Terfenadine. F: Pimozide. G: Mefloquine. H: Ellipticine. I: Trifluoperazine. Based on pkCSM ADMET predictive model [Ref: 24], a compound is said to have high Caco-2 permeability at a value of > 0.90; poor GIA at less than 30% absorption; low skin permeability (logKp > -2.5); VDss is low at < 0.71 L/kg (log VDss < -0.15) and high at > 2.81 L/kg (log VDss > 0.45); BBB permeant at a logBB > 0.33 and non-permeant at logBB < -1; CNS permeant at a logPS > -2 and non-permeant at a logPS < -3; *Tetrahymena pyriformis* toxicity (pIGC50) at a value > − 0.5 log µg/L is considered toxic; minnow toxicity (LC50) at a value < 0.5 mM (logLC50 < -0.3) is regarded as high acute toxicity; maximum recommended tolerated doses (MRTD) of ≤ 0.477 log(mg/kg/day) is considered low, and high if > 0.477 log(mg/kg/day)

The results of target prediction indicate the molecular targets which include: serotonin receptor 2a/2b/2c, HERG protein, adrenergic receptor alpha-1a/2a, dopamine D3 receptor, nitric oxide synthase, inducible (iNOS), adrenergic receptor alpha-1d/beta-1, carbonic anhydrases, epidermal growth factor receptor erbB1 (EGFR), tyrosine-protein kinases, norepinephrine transporter, and C-C chemokine receptor type 5 (CCR5); these targets cut-across only nine drug compounds (dexverapamil, emetine, parthenolide, dobutamine, terfenadine, pimozide, mefloquine, ellipticine and trifluoperazine), at a threshold of 20% probability (Table 3).

**Table 3:** Results of molecular targets prediction

SN	Ligands	% Probability of predicted targets																																
		A	B	C	D	E	F	G		H		I		J		K		L		M		*N*		O		*P*		Q			*R*		S	
1	Dexverapamil	100	100	100	100																													
2	Emetine		58			40																												
3	Parthenolide						63		63																									
4	Dobutamine			100	100						100		100		100		100		100															
5	Terfenadine	100	100	100					100								100		100		100		52											
6	Pimozide		98		98																				98		98							
7	Mefloquine		100																								90		98					
8	Ellipticine																													100				
9	Trifluoperazine	35	100	33	100														40				26									100		

Serotonin receptor 2a/2b/2c (HTR2A/HTR2B/HTR2C, P28223/ P41595/P28335). B: HERG (KCNH2, Q12809). C: Adrenergic receptor Alpha-1a/Alpha-2a (ADRA1A /ADRA2A, P35348/P08913). D: Dopamine D3 receptor (DRD3, P35462). E: Small conductance calcium-activated potassium channel protein 1/2/3 (KCNN1/KCNN2/KCNN3, Q92952/Q9H2S1/Q9UGI6). F: Cyclooxygenase-2 (PTGS2, P35354). G: Nitric oxide synthase, inducible (by homology) (NOS2, P35228). H: Adrenergic receptor Alpha-1d/beta-1 (ADRA1D/ADRB1, P25100/P08588). I: Carbonic anhydrase I, II, III, IV, VA, VB, VI, VII, IX, XII, XIII (CA1/CA2/CA3/ CA4/ CA5A/ CA5B/ CA6/ CA7/ CA9, CA12, CA13, P00915/ P00918/ P07451/ P22748/ P35218/ Q9Y2D0/ P23280/ P43166/ Q16790/ O43570/ Q8N1Q1). J: Epidermal growth factor receptor erbB1 (EGFR, P00533). K: Tyrosine-protein kinase FYN/LCK (FYN/LCK, P06241/ P06239). L: Norepinephrine transporter (SLC6A2, P23975). M: C-C chemokine receptor type 5 (CCR5, P51681). N: Muscarinic acetylcholine receptor M1/M2/M4 (CHRM1/CHRM2/CHRM4, P11229/P08172/P08173. O: Ubiquitin carboxylterminal hydrolase 1 (USP1, O94782). P: Glycine receptor subunit alpha-1 (GLRA1, P23415). Q: Adenosine receptor A1,A2a, A2b, A3 (ADORA1, ADORA2a, ADORA2b ADORA3, P30542/P29274/ P29275/P0DMS8). R: DNA topoisomerase II alpha (TOP2A, P11388). S: Anti-estrogen binding site (AEBS) (EBP, Q15125)

**Fig. 6:**
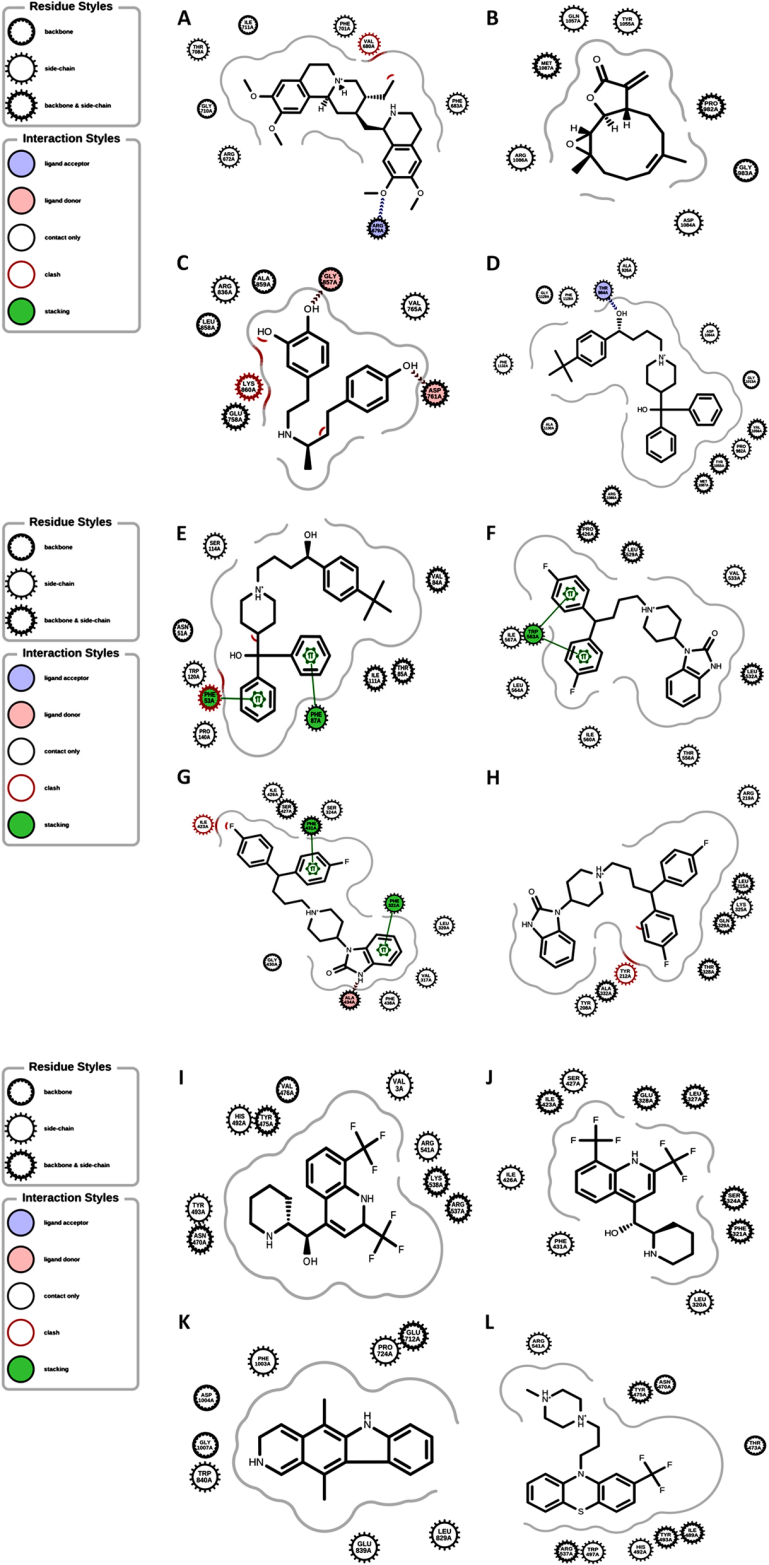
Binding interaction of **A** Emetine and HERG. **B** Parthenolide and Nitric oxide synthase, inducible. **C** Dobutamine and Epidermal growth factor receptor erbB1. **D** Terfenadine and inducible nitric oxide synthase. **E** Terfenadine and Tyrosine-protein kinase FYN. **F** Pimozide and HERG. **G** Pimozide and Glycine receptor subunit alpha-1. **H** Pimozide and Dopamine D3 receptor. **I** Mefloquine and HERG. **J** Mefloquine and Glycine receptor subunit alpha-1. **K** Ellipticine and DNA topoisomerase II alpha. **L** Trifluoperazine and HERG

Molecular docking was conducted on selected seven molecular targets, and the results in Table 4, showed that highest binding affinity occurred between Pimozide and Dopamine D3 receptor (-8.035 kcal.mol^−1^), followed by Terfenadine binding to inducible nitric oxide synthase (-7.833 kcal.mol^−1^), Pimozide binding to Glycine receptor subunit alpha-1 (-7.740 kcal.mol^−1^), and Pimozide binding with HERG (-7.636 kcal.mol^−1^). The selected docking poses of the ligand-protein complexes are presented in Fig. 6.

**Table 4:** Molecular docking binding affinity of ligand-protein interaction

SN	Ligands	Binding Affinity ΔG (kcal.mol^−1^)						
		HERG (AF-Q12809)	DRD3 (AF-P35462)	NOS2 (AF-P35228)	EGFR (AF-P00533)	FYN (AF-P06241)	GLRA1, (AF-P23415)	TOP2A (AF-P11388)
1	Dexverapamil	-5.924	-4.649					
2	Emetine	-7.044						
3	Parthenolide			-7.041				
4	Dobutamine		-5.152		-6.105	-6.640		
5	Terfenadine	-6.181		-7.833		-6.814		
6	Pimozide	-7.636	-8.035				-7.740	
7	Mefloquine	-7.217					-7.068	
8	Ellipticine							-7.396
9	Trifluoperazine	-7.335	-6.537					

Gene name (gene code, UniProt ID) **-** B: HERG (KCNH2, Q12809). D: Dopamine D3 receptor (DRD3, P35462). I: Nitric oxide synthase, inducible (by homology) (NOS2, P35228). L: Epidermal growth factor receptor erbB1 (EGFR, P00533). M: Tyrosine-protein kinase FYN (FYN, P06241). T: Glycine receptor subunit alpha-1 (GLRA1, P23415). V: DNA topoisomerase II alpha (TOP2A, P11388). Docking parameters: B: HERG [spacing: 1.000, npts: 126 × 126 × 126, center: -6.474 × 3.692 × -0.454]. D: Dopamine D3 receptor [spacing: 0.800, npts: 98 × 80 × 126, center: -8.214 × -1.817 × 7.114]. I: Nitric oxide synthase, inducible (by homology) [spacing: 0.750, npts: 126 × 126 × 126, center: -4.707 × -0.769 × 3.509]. L: Epidermal growth factor receptor erbB1 [spacing: 0.800, npts: 126 × 126 × 126, center: -6.474 × 3.692 × -0.454]. M: Tyrosine-protein kinase FYN [spacing: 0.525, npts: 126 × 126 × 126, center: -4.721 × 3.182 × -0.374]. T: Glycine receptor subunit alpha-1 [spacing: 0.750, npts: 126 × 126 × 126, center: -8.842 × 0.109 × 5.951]. V: DNA topoisomerase II alpha [spacing: 1.000, npts: 126 × 126 × 126, center: 5.135 × -1.990 × 7.409]

**Fig. 7:**
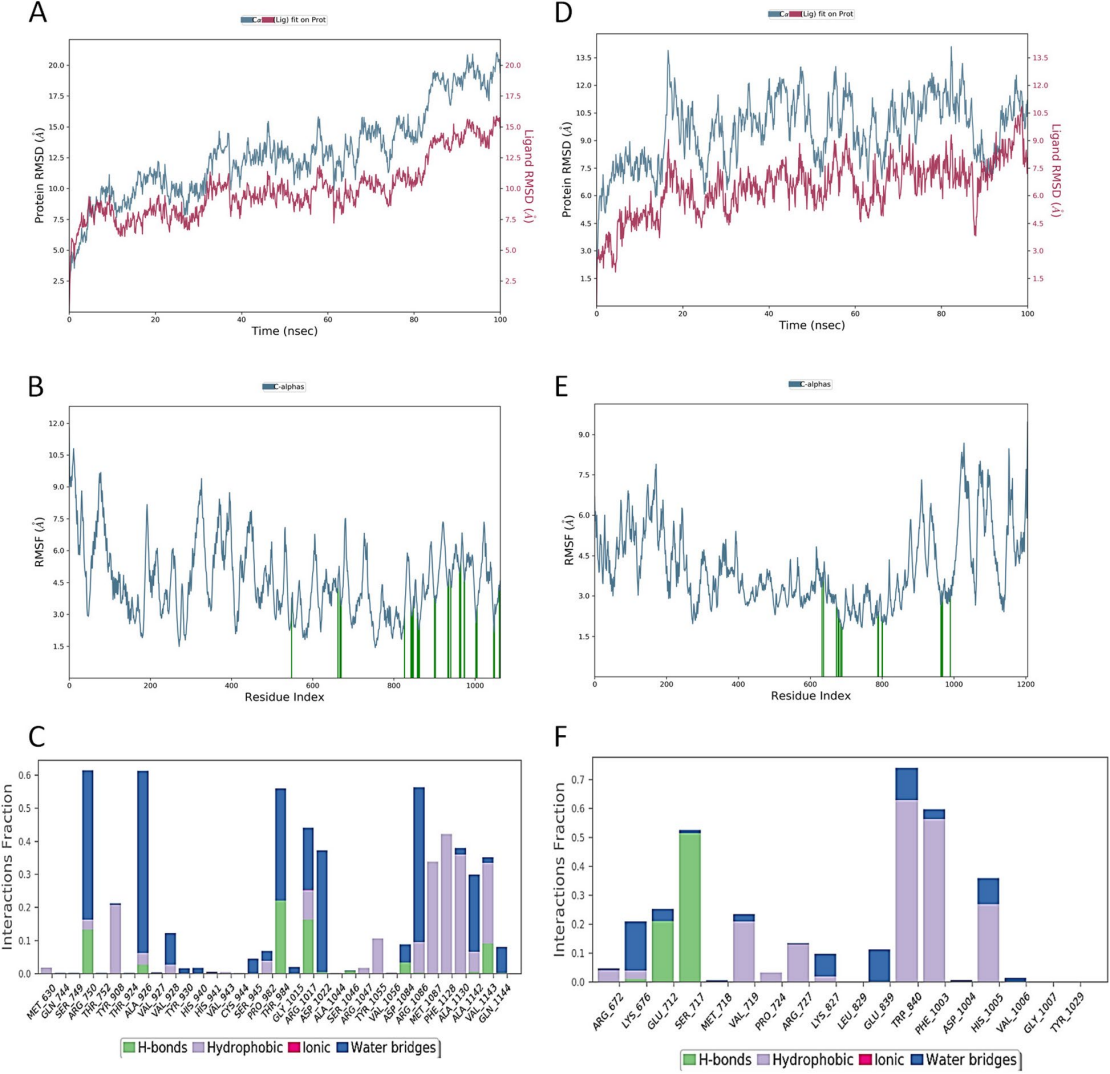
Molecular dynamic simulation (MDS) results. **A** RMSD of Terfenadine and inducible nitric oxide synthase (iNOS). **B** RMSF of iNOS. **C** Interaction profile of the contact between Terfenadine and inducible nitric oxide synthase. **D** RMSD of Ellipticine and DNA topoisomerase II alpha. **E** RMSF of DNA topoisomerase II alpha. **F** Interaction profile of the contact between Ellipticine and DNA topoisomerase II alpha

The results of molecular dynamic (MD) simulations are presented in Fig. 7. The results of RMSD of Terfenadine-iNOS complex indicated RMSD of 20.00 Å (Fig. 7A), RMSF of iNOS showed broad fluctuation in the amino acid residues (Fig. 7B), and protein-ligand interactions (or contacts) are presented in Fig. 7C, indicate various amino acid residues that formed hydrogen bonds, hydrophobic, ionic and water bridges. The results of Ellipticine-TOPIIα complex indicated RMSD of 13.50 Å (Fig. 7D). the RMSF of TOPIIα occurred maximally at both N-and C- terminal (Fig. 7E), and the protein-ligand interactions (or contacts) are presented in Fig. 7F, which indicate various amino acid residues that formed hydrogen bonds, hydrophobic, ionic and water bridges.

Overall, the results of protein-ligand interactions validated the amino acid residues present in the docking interactions of Terfenadine-iNOS complex and Ellipticine-TOPIIα complex. The binding free energies of the two complexes were calculated using MMGBSA at 0 ns and 100 ns respectively and the results are shown in Table 5. Overall, binding energy ΔG^bind^ (Total) at 0 ns was lower than that of 100 ns for both the Terfenadine-iNOS complex (-101.707 to -103.302 kcal.mol^−1^) and Ellipticine-TOPIIα complex (-42.229 to -58.780 kcal.mol^−1^). These results showed that the two complexes were energetically favourable during the simulation condition.

**Table 5:** Prime MMGBSA binding energy of Terfenadine-iNOS complex and Ellipticine-DNA topoisomerase II alpha complex

Complex	Simulation Time (ns)	MMGBSA Binding energy ΔG (kcal.mol^-1^)							
		Coulomb	Covalent	Hbond	Lipo	Packing	Solv_GB	vdW	ΔG^bind^ (Total)
Terfenadine-iNOS.	0	-10.657	2.512	-0.104	-56.242	-0.824	24.980	-61.371	-101.707
	100	-8.908	1.585	-0.8252	-61.007	-0.714	30.745	-64.180	-103.302
Ellipticine-TOPIIα	0	-6.674	1.262	-0.265	-23.614	-0.057	16.593	-29.472	-42.229
	100	-12.789	-0.059	-0.250	-26.857	-0.543	17.257	-35.537	-58.780

*Legend*: Total: Total energy (Prime energy). Coulomb: Coulomb energy. Covalent: Covalent binding energy. Hbond: Hydrogen bonding energy. Lipo: Lipophilic energy. Packing: Pi-pi packing correction. Solv GB: Generalized Born electrostatic solvation energy. vdW: Van der Waals energy

## Discussion

Prostate cancer (PCa) is a complex and biologically diverse disease [5]. In this study, the differentially expressed genes (DEGs) implicated in PCa were examined. Downregulated DEGs play a significant role in the disease progression. Enriched kinases such as MAPK14, MAPK1, MAPK13, CSNK2A1, CDK1, CDK4, ERK1, ERK2, and DNAPK are involved in signaling pathways regulating cell growth, proliferation, and survival. Transcription factors like STAT3, TCF3, SRF, SUZ12, NFE2L2, SMAD4, SOX2, GATA2, and EGR1 are critical in controlling gene expression associated with PCa development and progression. Intermediate proteins like SP1, GSK3B, RELA, JUN, RB1, HDAC3, SIRT1, and NCOR2 modulate various cellular processes contributing to PCa pathogenesis. Moreover, several molecular pathways have been reported involved in PCa include the regulation of AR activity by gene fusion events involving BMI, ERG, FOXA1, MAGI2, MAP3K7, MYC, NKX3.1, TP53, SMAD4, SOX9, and various signaling pathways [5]. Understanding the roles of these molecules can provide insights into the molecular mechanisms underlying PCa and potentially identify new therapeutic targets.

The X2K approach can assist in drug target discovery and help in unraveling drug mechanisms of action. As a limitation, currently the X2K method uses only protein/DNA interactions, protein–protein interactions and kinase–substrate reactions, other types of data could be added [22]. Another limitation of the X2K method is the assumption of independence between regulators and targets when applying the enrichment analyses [22]. Moreover, X2K method has been successfully used to investigate network analysis of DEGs associated with myeloproliferative disorders [37]. Connectivity Map [38] which utilize gene-expression signatures to connect small molecules, genes and disease, has been used to identify pimozide as promising drug against cabazitaxel-resistance in CRPC [39]. The ligands identified in this study cover a wide range of mechanisms that could potentially reverse gene expression changes in PCa. Talampicillin, dexverapamil, gemfibrozil, dobutamine, and ginkgolide A are known for their roles in modulating pathways related to cancer progression or treatment resistance. Similarly, drugs like parthenolide, emetine, and cycloheximide have been studied for their ability to inhibit cancer cell growth and induce apoptosis. On the other hand, drugs like terfenadine, camptothecin, and trichostatin A are associated with reversing downregulated genes in PCa possibly by affecting pathways involved in tumor suppression or DNA repair through the predicted protein targets, and associated kinases and transcription factors.

In comparison to existing PCa therapies, nine compounds identified in this study have potential advantages and disadvantages in term of their mechanisms of action, side effect profiles, and the current landscape of PCa treatment. *Dexverapamil* is known for its ability to inhibit P-glycoprotein, which is often implicated in multidrug resistance (MDR) reversal, potentially enhancing the efficacy of chemotherapy [40]. Being a calcium channel blocker, it might offer cardioprotective benefits, potentially useful in patients with concurrent cardiovascular conditions. Dexverapamil exhibited improved potency but failed to proceed to clinical application because of its unwanted interactions with the CYP450 enzymes leading to unfavorable pharmacokinetic profiles [41]. *Emetine* is a metabolite from the root of *Carapichea ipecacuanha* (Brot.) [42]. It is a ribosomal and mitochondrial protein synthesis inhibitor, as well inhibits the synthesis of RNA and DNA. Emetine is known to induce apoptosis in cancer cells by downregulation of anti-apoptotic and upregulation of pro-apoptotic gene products in various cancer cells including in PCa [43, 44]. It could provide antiviral and antiparasitic properties as additional benefits if the patient has concurrent infections. However, its has toxicity profile requires further investigation.

*Parthenolide* is a major active component of the medicinal plant *Magnolia grandiflora* and *Tanacetum parthenium*), which is conventionally used to treat inflammatory diseases such as fever, migraine, and arthritis [45]. It has multi-targets mechanism against cancer [46]. Parthenolide is an inhibitor of NF-κB, that also inhibit several cytokines, including tumor necrosis factor-α, RANKL, and interleukin-1β [47]. The radiosensitization effect of parthenolide in PCa cells is mediated by nuclear factor-κB inhibition [48]. A study has shown that parthenolide sensitises prostate tumour tissue to radiotherapy while protecting healthy tissues [49]. It has anti-inflammatory properties, thus help to manage inflammation associated with cancer [46, 50]. However, it possesses poor water solubility and bioavailability, making it difficult to deliver effective doses. There is more preclinical than clinical evidence, so its efficacy in humans is not well-established.

*Dobutamine* is a β1-adrenergic agonist used to support heart function, which could be beneficial for PCa patients with heart failure [51]. Dobutamine inhibits the yes-associated protein (YAP)-dependent gene transcription, which has been observed in a number of types of tumors [52]. Dobutamine has been reported for significantly inhibit proliferation, increase apoptosis, induce expression of caspases 3 and 9, arrest the cell cycle at the G2/M transition stage, and reduce migration and invasion of MG-63 osteosarcoma cells in a time- and concentration-dependent manner, thereby [53]. *Terfenadine* is a histamine receptor antagonist like cimetidine, which could help with cancer symptoms such as histamine-related inflammation or pruritus. It has been suggested that inhibition of histamine h3 receptor (H3R) may have favorable application prospects in the treatment of PCa [54]. A study has shown that terfenadine induces anti-proliferative and apoptotic activities in human hormone-refractory PCa through histamine receptor-independent mechanism [55]. Also, it has been suggested that terfenadine induces the DNA damage response in human melanoma cells [56].

*Pimozide* has antipsychotic properties which can manage psychiatric symptoms in cancer patients, such as anxiety or delirium. It is evident based on research reports that pimozide could inhibit invasion and migration of cancer cells [57]. In mice, pimozide reduced the progression of PCa with increased reactive oxygen species (ROS) generation and decreased superoxide dismutase I (SOD1) activity [57]. Pimozide has been identified as a promising candidate drug for cabazitaxel-resistant CRPC, where AURKB and KIF20A were found as potential targets [39]. Phosphorylated STAT3 (Tyr705) has been identified as a biomarker of response predictive of sensitivity to pimozide treatment in triple-negative breast cancer [58]. In the context of PCa, pimozide has been shown to inhibit cell growth through the suppression of STAT3 activation [59]. These findings suggest that targeting STAT3 signaling pathway may hold therapeutic potential in PCa treatment.

*Mefloquine* is an antimalarial compound with anticancer potential [60]. A previous experimental study has shown that mefloquine at 20 µM selectively and completely abolished the cell proliferation of two human PCa cell lines DU145 and PC3, by hyperpolarization of mitochondrial membrane potential and increased production of ROS resulting in rapid cancer cell death through inhibition of Akt phosphorylation and activated JNK, ERK and AMPK signaling [60–62].

*Ellipticine* is metabolite present in a medicinal plant *Ochrosia elliptica* labil, with mechanism of action that involve intercalation into DNA, inhibiting topoisomerase II, which is a promising mechanism for cancer treatment [63]. Ellipticine and its derivatives have shown activity against various cancer types, potentially including PCa [64]. However, it is hepatotoxic and has inconsistent absorption and metabolism which can complicate dosing and limits its clinical use. *Trifluoperazine* is antipsychotic with potential anticancer effects. Some studies suggest it may inhibit cancer cell proliferation, induce apoptosis and overcomes drug resistance [65]. It has been reported that trifluoperazine effectively inhibited cisplatin-resistant metastatic bladder urothelial carcinoma and circumvented cisplatin resistance with concurrent Bcl-xL downregulation [66]. However, its neurological side effects such as extrapyramidal symptoms and tardive dyskinesia worth further investigation.

In PCa, the factors such as (i) low gastrointestinal absorption (GIA) can affect drug bioavailability, influencing its effectiveness, (ii) Inhibitors of cytochrome P450 enzymes, particularly CYP1A2, CYP2C19, CYP2C9, CYP2D6, and CYP3A4, can alter the metabolism of drugs used in PCa treatment, impacting their pharmacokinetics and potentially therapeutic outcomes. (iii) Additionally, being a substrate of P-glycoprotein can affect drug distribution and elimination, influencing its concentration in prostate tissue. Avoiding inhibitors of P-glycoprotein can help maintain optimal drug levels. (iv) AMES toxicity and inhibiting hERG I and II can minimize potential adverse effects on genetic material and cardiac function, respectively, enhancing the safety profile of the treatment regimen. (v) Avoiding drugs with potential hepatotoxicity is crucial in PCa management to prevent liver damage, especially considering the importance of liver function in drug metabolism and clearance.

Currently, there are approximately 25 drug targets under investigation for the treatment of PCa, including androgen receptor (AR), AR cofactors and regulators (such as NCOA1, NCOR1, TNK2, and others), androgen synthesis enzymes (e.g., CYP17), aurora A kinase, cyclin-dependent kinases (CDKs), growth factor receptors (EGFR, IGF1R, FGFR, VEGFR, MET), and tyrosine kinase (SRC) [5]. In this study, several molecular targets suitable for therapeutic purposes were identified. These targets include the serotonin receptors, HERG protein, dopamine D3 receptor, inducible nitric oxide synthase (iNOS), Norepinephrine transporter, epidermal growth factor receptor erbB1 (EGFR), tyrosine-protein kinases, glycine receptor subunit alpha-1, and DNA topoisomerase II alpha. Serotonin signaling may influence PCa growth and metastasis through these receptors. Targeting serotonin receptors could potentially modulate tumor behavior. HERG (Human Ether-à-go-go-Related Gene) encodes a potassium channel protein. Dysregulation of HERG channels has been implicated in cancer development, including PCa. Targeting HERG may affect tumor cell proliferation and survival. Carbonic anhydrases regulate pH homeostasis in tumor microenvironments. In PCa, targeting carbonic anhydrases could disrupt tumor acidification and inhibit metastasis. Norepinephrine signaling contributes to PCa progression. Inhibiting the norepinephrine transporter may interfere with tumor cell proliferation and invasion. Chemokine receptors, including CCR5, play roles in cancer cell migration and metastasis. Blocking CCR5 signaling could potentially inhibit metastasis of PCa.

EGFR signaling is dysregulated in various cancers, including PCa. Inhibiting EGFR could potentially suppress tumor growth and invasion. Furthermore, kinase activities of EGFR, ephrin type-A receptor 2 (EPHA-2), JAK2, ABL1, and SRC were found to be increased in PCa based on phosphotyrosine peptide enrichment analysis [5, 67]. The IL6-IL6R signaling pathway, leading to activation of the JAK1-STAT3 pathway, is also involved. STAT3 interacts with AR and facilitates recruitment of p300 to the AR transcriptional complex [5, 68, 69]. Extracellular growth factors such as EGF, IGF, FGF10, and others, can transactivate AR through engagement with receptor tyrosine kinases (RTKs), which in turn activate the PI3K and MAPK pathways [5]. EGFR, in particular, is frequently overexpressed in many cases of PCa [5, 70].

Protein kinases are enzymes that phosphorylate and transfer a phosphate group from ATP to specific residues like tyrosine, serine, or threonine. Tyrosine kinases are involved in various cellular processes and are often dysregulated in cancer. Targeting specific tyrosine kinases could disrupt oncogenic signaling pathways in PCa cells. Tyrosine kinase inhibitors (TKIs) such as Sorafenib and erlotinib have been developed for the treatment of various cancers [71]. Mutations in the kinase domain of the epidermal growth factor receptor (EGFR) are known oncogenic drivers. TKIs targeting mutated EGFR have shown superior efficacy compared to chemotherapy in treating patients with EGFR-positive cancer and have become the standard of care [72]. The MAP kinase signaling pathway has been identified as significant in the metastatic process, and its involvement in androgen receptor signaling has been previously described [73]. Muscarinic receptors consist of five distinct subtypes (M1-M5), and their localization studies suggest that multiple subtypes (M1, M3, M4, and M5) are expressed in pancreatic islets, -cells, or -cell derived tumor cell lines [74].

Moreover, topoisomerase II alpha (Topo IIα) was identified as one of the protein targets. Inhibition of Topo IIα has been suggested as a potential therapeutic option against CRPC, which has link with androgen independence in cellular growth [75–77]. Topo IIα is known to promote tumor aggressiveness by inducing chromosomal rearrangements of genes that contribute to a more invasive phenotype in PCa cells. It also enhances the androgen receptor signaling pathway by facilitating the transcription of androgen-responsive genes. Additionally, Topo IIα expression is significantly higher in cabazitaxel-resistant CRPC cells compared to cabazitaxel-sensitive CRPC cells, suggesting that inhibiting Topo IIα could be a viable therapeutic strategy for CRPC [78, 79]. Also, in this study we identified inducible nitric oxide synthase (iNOS) as a key protein target. Nitric oxide (NO), which plays complex roles in cancer, including PCa. NO has been implicated in androgen resistance, with studies suggesting its involvement in androgen receptor transcriptional suppression and direct androgen receptor inhibition through iNOS and endothelial nitric oxide synthase (eNOS), respectively [80, 81]. High iNOS expression in the tumor epithelium of the prostate has been associated with lethal disease, and epigenetic changes and polymorphisms in the iNOS gene are correlated with an increased risk of PCa development, suggesting the involvement of iNOS in prostate carcinogenesis [82]. Thus, inhibition of iNOS and eNOS may contribute to anti-cancer effects.

In cancer therapy, drug combination approach has been found to overcome the problems related to monotherapy and several studies have already demonstrated the superiority of combined therapies compared to monotherapy [83]. Combinations of small molecular inhibitors against specific DNA repair proteins and cytotoxic drugs have been suggested as future approach to achieve success in cancer treatment [84]. The identified drugs could have potential interactions with existing PCa treatments. For synergistic effects; *Dexverapamil* could enhance the efficacy of chemotherapeutic agents like docetaxel or mitoxantrone by inhibiting P-glycoprotein and reversing multidrug resistance (MDR). Also, combining Dexverapamil with androgen deprivation therapy (ADT) might improve outcomes by sensitizing cancer cells to hormone depletion. *Emetine’s* apoptosis-inducing effects might be synergistic with drugs like bicalutamide, which also promote apoptosis in PCa cells. It could be combined with chemotherapeutics to enhance cytotoxic effects through protein synthesis inhibition and apoptosis induction. *Parthenolide’s* inhibition of NF-κB could be enhanced by combining it with other NF-κB pathway inhibitors, potentially leading to reduced cancer cell proliferation and survival. Its anti-inflammatory properties might work synergistically with drugs that also target inflammation, reducing tumor-promoting inflammation.

Although, *dobutamine* is not a direct anticancer agent, dobutamine could be used to manage cardiac side effects of existing cancer therapies, allowing for higher tolerable doses of those therapies. *Terfenadine* itself poses risks, but exploring safer histamine receptor antagonists in combination with standard treatments might help manage cancer-related inflammation and histamine-mediated effects. *Pimozide’s* ability to inhibit cancer cell migration and invasion could be combined with agents that target metastatic pathways, providing a multi-pronged approach to preventing metastasis. Combining pimozide with other psychotropic drugs might help manage psychological symptoms in PCa patients. Since *mefloquine* disrupts lysosomal function, it could be combined with autophagy inhibitors to enhance cancer cell death. Mefloquine might sensitize cancer cells to chemotherapy, potentially lowering required doses and reducing side effects. Combining *ellipticine* with other DNA-damaging agents like platinum-based drugs could enhance the overall DNA damage, leading to increased cancer cell death. Synergistic effects might be explored with other topoisomerase inhibitors to enhance anti-cancer efficacy. *Trifluoperazine’s* inhibition of calmodulin could be combined with other agents affecting calcium signaling pathways to disrupt cancer cell growth [85]. Using it alongside other antipsychotics might help manage neurological symptoms in cancer patients.

Molecular docking is a computational technique used to predict the preferred orientation of one molecule (the ligand) when bound to another molecule (the target, typically a protein) to form a stable complex [18]. Binding affinity have biological implications on drug efficacy and potency. Low binding affinity indicates strong binding between the ligand and the target, suggesting that the ligand is likely to be a potent inhibitor or activator of the target [26]. The lower (more negative) the binding energy, the stronger the interaction. High binding energy suggests weak interactions, implying that the ligand is less likely to be effective in modulating the target’s activity. Moreover, docking provides hypotheses that need to be confirmed through biochemical assays, crystallography, or other biophysical methods. Discrepancies between predicted and observed binding affinities highlight the limitations of current docking methods and the need for continuous refinement.

MD simulation helps simulate the movement of atoms and molecules over time, providing insights into protein dynamics and behavior, and articulate on the stability of the protein–ligand complex in a simulated condition [26, 86]. In MD simulations, the stability and binding affinity of a protein-ligand complex are often assessed using various metrics which include Root Mean Square Deviation (RMSD), Root Mean Square Fluctuation (RMSF), and Molecular Mechanics Generalized Born Surface Area (MMGBSA) are commonly employed. The RMSD results indicate that Terfenadine-iNOS complex was less stable than Ellipticine-DNA topoisomerase II alpha complex. RMSD of about 2.0 Å indicates that the proteins had undergone relatively small conformational changes and were, thus, stable during the simulation [87]. RMSD measures the average deviation of a set of atomic positions (typically the backbone or all heavy atoms of the protein) from a reference structure over time. A lower RMSD indicates that the structure of the protein (or protein-ligand complex) remains closer to the initial or reference structure, suggesting greater stability. Monitoring RMSD over time helps in identifying significant conformational changes. Large deviations may indicate flexibility or instability. RMSF measures the average fluctuation of each atom or residue around its average position over the course of the simulation. RMSF provides insights into the flexibility of individual residues or regions within the protein. Higher RMSF values indicate greater flexibility. Regions with low RMSF in the protein-ligand complex might correspond to stable interaction sites, while regions with high RMSF might suggest flexible or less stable binding regions.

MMGBSA (Molecular Mechanics Generalized Born Surface Area) generates a lot of energy properties which report energies for the ligand, receptor, and complex structures as well as energy differences relating to strain and binding, and are broken down into contributions from various terms in the energy expression [26, 36]. The binding free energy (total) clearly showed that the stability of the complexes in physiological condition, and they were found to be reasonably stable. MMGBSA is a method to estimate the free energy of binding between a protein and a ligand by combining molecular mechanics energies with solvation terms (Generalized Born and Surface Area terms). MMGBSA calculates the free energy of binding, ΔG_bind_, which is an indicator of binding affinity. A more negative ΔG_bind_ suggests a stronger binding affinity. MMGBSA can be decomposed to analyze the contribution of individual residues to the binding free energy, identifying key residues involved in the binding process. By combining these analyses, a comprehensive understanding of the stability and binding affinity of the protein-ligand complex could be estimated.

### Implications of the study for personalized medicine

The implications of this study for personalized medicine in PCa include (1) Targeted therapy approach which involve identifying specific biomarkers in patients that predict responsiveness to these compounds, allowing for personalized treatment plans [88]. Also, the use of genetic profiling to tailor treatments based on individual tumor characteristics, improving efficacy and minimizing side effects. (2) Combination therapy which involve development of personalized combination therapies based on the patient’s unique genetic and molecular profile, enhancing treatment outcomes [89, 90]. (3) Drug repurposing which involve reapplication of existing drugs like the identified compounds can expedite the development of new treatments, leveraging existing safety data and potentially reducing costs and time to clinical use [19, 91].

### Limitations of the study and future perspective

The findings are primarily based on computational models and predictions, although there are few empirical data to confirm the efficacy and safety of these compounds in cancer treatment. Computational predictions may overestimate the therapeutic potential due to the complexity of biological systems that are not fully captured by models. Also, potential off-target effects and toxicities identified computationally may not fully predict the in vivo outcomes, leading to unforeseen adverse effects. Addressing limitations in future research, there will be need to conduct in vitro and in vivo studies on PCa cell lines to assess the compounds’ efficacy and elaborate the mechanisms of action, as well as to evaluate the pharmacokinetics, pharmacodynamics, and toxicity profiles. There is need to initiate phase I clinical trials to assess safety, dosing, and initial efficacy in humans, as well as design trials that investigate combinations of these compounds with existing therapies to explore synergistic effects. Furthermore, advanced computational tools such as machine learning and artificial intelligence (AI), could be used to refine predictions and identify potential synergistic combinations with greater accuracy.

## Conclusion

This study has unravelled from the DEGs of PCa patient, the potential drugs for repurposing pharmacological indication. The identified compounds (Dexverapamil, Emetine, Parthenolide, Dobutamine, Terfenadine, Pimozide, Mefloquine, Ellipticine, and Trifluoperazine) present promising mechanisms of action involving modulation of HERG, adrenergic receptor Alpha-1a, dopamine D3 receptor, epidermal growth factor receptor erbB1, C-C chemokine receptor type 5, muscarinic acetylcholine receptors, glycine receptor subunit alpha-1 and others, which could potentially enhance PCa treatment. Combinatorial therapy involving these drugs or in addition with existing standard PCa therapies, could provide synergistic effects by reversing multidrug resistance, inducing apoptosis, inhibiting metastasis, and modulating critical signaling pathways. However, their off-target effects and toxicity profiles necessitate careful consideration and further investigation. Experimental validation and clinical trials are crucial next steps to confirm their therapeutic potential and safety. Integrating these findings into personalized medicine approaches could revolutionize PCa treatment, providing more effective and tailored therapeutic options for patients. Future research should focus on translating these insights into clinical practice, ultimately improving patient outcomes and quality of life.

## Data Availability

All data associated with the current study are. included in this article. This can also be found at fuoye.edu.ng after publication as well as on this journal website.
